# Translations

**Published:** 1882-06

**Authors:** Seth N. Jordan

**Affiliations:** Columbus, Ga.


### Translations.

BY SETH N. JORDAN, M.D., COLUMBUS, GA.

*From Foreign Journals for the Atlanta Medical Register.*

*Bacteria of Tuberculosis.*—Up to a recent date the only forms of bacteria spoken of, casually, by a few authors (*Klebls, Schuller*) were micrococci and cylindrical or spherical bacteria. Very recently Aufrecht has written a communication in which he asserts that the centre of the miliary tubercle does not consist, as is generally accepted, in detritus, but is filled with micro organisms, "very small micrococci, then rows and links of two or three together, and, finally, bright, short, staff-like bodies, which are only one half longer than the diameter measures." Since Aufrecht's communication was published Prof. Baumgarten, in Konigsberg, almost simultaneously with Reg. Rath Koch, in Berlin, have made public their investigations (*Centralblatt f. Med. W., and Berlin Klin. Wochen.*) in this direction. Baumgarten succeeded, not only in finding the bacteria in animals infected internally with tuberculosis, but also in tubercles in man. These bacteria resemble the bacterium termo, being, however, longer, more slender and instead of rounded ends they possess knotty, bulbous terminations. They rarely go in pairs, and never group themselves in the form of zooglœa. Again, they are distinguishable from bacteria of decomposition and other species of bacteria in that Weigert's fluid, with the aid of Koch's method of illumination, does not render them distinct. Baumgarten recommends the following method of treating the preparation before it be microscopically examined: Place the fresh preparation containing the tubercles in absolute alcohol, to remain 24 hours, then the sections are put into a weak solution of natrium or kalium lye, to remain until clear. Baumgarten's bacteria are found not only in the centre of the tubercle but over the entire periphery. Each staff is five or six times as long as broad. These bacteria do not occur in conjunction with either cocci or diplococci.

*Intra-Arterial Infusion of Salt in Hemorrhage. Wochensch.* 1881. *No.* 51.—Patient was delivered, in the hospital at Basle, by forceps, of a dead child, had fever before labor set in, the placenta was adherent and was extracted manually. Immediately before this last was accomplished violent hemorrhage took place. Pulse, 156; respirations, 42; extremities cold; very restless, and death seemed imminent. A 8.6 per cent solution of common salt, with the addition of a few drops of potash lye, was infused into the right radial artery. During the infusion patient improved; the pulse sank to 112; later, to 108; the restlessness disappeared, and patient recovered in spite of beginning perimetritis, and later, mastitis. Kronecker and Sander have recently discovered, in the case of animals in syncope from loss of blood, that it is unnecessary to add any potash in solution.

---

*Prevention of Inflammation of the Eyes in Infants. Crede. Arch. fur Gyn., XVIII, p.* 367.—In connection with a former publication on this subject, Crede communicates 400 more cases in which, immediately after birth, he washed out the eyes with pure water and dropped a single drop of a two per cent. solution of argent. nitrici, by means of a glass rod, into each eye. Crede no longer uses bandages dipped in salicylic acid solution. Not a single one of the children thus treated was attacked with ophthalmia neonatorum. Therefore C. recommends that every midwife be advised to keep on hand constantly a two per cent. sol. of argent. nitr. and a glass rod.

---

*Neuralgia of the Trigeminus Cured by Amputation of the Portio Vaginalis. Holst. Wochenschr. No.* 1. 1882.—Patient was attacked with dysmenorrhœa after bathing, during menstruation, in cold water. On visiting Kruznach this was cured up. In its stead a neuralgia of the second and third branches of the trigeminus set in, which grew worse at each menstruation. H. found the portio vaginalis of the uterus much swollen and indurated—a case of endometritis colli follicularis. After amputation colli the neuralgia disappeared; the first menstruation was not without pains, however; the second was without uterine pain or neuralgia.

---

*Electricity in the Treatment of Affections of the Joints. Joffroy. Centralblat', f. Med. Wissensch. No* 16. 1882.—When acute inflammatory phenomena are present no electricity should be applied. (Only the constant current is considered). Especially useful is the electric current in affections of the joints following traumata and affections arising during the lying-in period and from gonorrhœa. Here, in many cases, the treatment can be directed to the extra articular lesions (atrophy of muscles, and hypertrophy of the fibrous tissue). In gout and chronic rheumatism electricity is almost always inert.

---

*Secondary Suture of the Ulnar Nerve. Berlin, Klin. Wochenschr.* 1881. *No.* 46.—Man, healthy, 27 years old; in consequence of a division of the right ulnar nerve just above the wrist; suffered for four months with a complete paralysis of this nerve. With absolute antiseptic precautions an operation was made, the nerve found and union accomplished by means of a catgut suture. Operation wound healed rapidly and completely. The result, in spite of careful after treatment, in regard to the restitution of the motor as well as sensible condition, remained negative.

---

*Condurango and Carcinoma. A. Hoffmann. Diss. Basel.* 1881. *Centralblatt, f. Med. Wissensch. No.* 15. 1882.—H. gives a tabulated list of all cases of carcinoma treated with condurango in the hospital at Basel, since 1871. For three years past all cases of cancer of the stomach and bowels have been treated exclusively with condurango. H.'s list embraces, in all, 132 cases. Treated with condurango, 20; of these 2 remain unimproved; improved, 8; died, 10. Treated without condurango, 108; unimproved, 28; improved, 10; died, 70.

---

*Treatment of Phthisis Laryngea. Beetz. Berlin Klin. Wochenschr.* 1882. *No.* 2.—B. recommends that phthisical laryngeal ulcers be sprinkled with powdered iodoform.

In this way, he believes. we can treat these ulcers antiseptically in truth, and in addition the specific anti-tuberculous effect of the iodoform is obtained. He makes from three to four insuffations each day.

*Grindelia Robusta for Asthma. Bronbelon. Wochenschr. No.* 48. 1881.—B. recommends cigarettes made of tobacco steeped in the alcoholic extract of grindelia robusta.

